# Evaluation of ELISA and Brucellacapt tests for diagnosis of human Brucellosis

**Published:** 2013-03

**Authors:** Hadi Peeridogaheh, Mohammad Ghasem Golmohammadi, Farhad Pourfarzi

**Affiliations:** 1 Department of Microbiology, School of Medicine, Ardabil University of Medical Sciences, Ardabil 56197, Iran; 2 Department of Anatomy, School of Medicine, Ardabil University of Medical Sciences, Ardabil 56197, Iran; 3 Department of Community Medicine, School of Medicine, Ardabil University of Medical Sciences, Ardabil 56197, Iran

**Keywords:** Brucellacapt, Brucellosis, ELISA

## Abstract

**Background and Objectives:**

Brucellosis is one of the most common zoonotic diseases in Iran and human brucellosis is endemic in all parts of the country. Because of the difficulty in the diagnosis of brucellosis, particularly in endemic areas, the use of new and feasible diagnostic tests seem to be of great importance for resolving the diagnostic obstacles. We evaluated the usefulness of a new serological test based on an immunocapture-agglutination technique in comparison with ELISA test for serological diagnosis of brucellosis.

**Materials and Methods:**

A total of 11 patients with brucellosis, who had positive blood cultures for *Brucella* species, and 47 suspected patients were included in this study. Serum samples collected from these patients were tested by brucellacapt and ELISA and the results were, consequently, compared.

**Results:**

In patients with positive blood culture, all the samples gave positive results with brucellacapt test while IgM ELISA, IgG ELISA and (IgG + IgM) ELISA tests were positive in 8, 9 and 11 patients, respectively. Out of the 46 suspected patients, (IgG + IgM) ELISA, Brucellacapt, IgG ELISA and IgM ELISA were positive in 37, 15, 34 and 37 patients, respectively.The best cut-off point of ELISA-IgG was 10.78 IU/ml which produced the maximal sensitivity and specificity for the diagnosis of human brucellosis.

**Conclusion:**

Both the (IgG + IgM) ELISA and Brucellacapt tests demonstrate a high specificity in this study. According to the results of the current study, it is found that both tests are valuable tools for diagnosis of brucellosis in Iran as an endemic area of brucellosis. It is strongly suggested that a combination of both tests to be used for the diagnosis of brucellosis.

## INTRODUCTION

Brucellosis is a major public health concern in developing countries. The disease is found worldwide, but is more common in the Mediterranean countries, the Arabian Peninsula, Indian subcontinent and in parts of Mexico and Central and South America (1, 2). In Iran, brucellosis represents a major health problem and is being reported with increasing frequency from various parts of the country. Brucellosis is quite a common disease throughout the country (3), the diagnosis of which is frequently difficult to establish. This is not only because the disease clinically mimics other infectious and non-infectious diseases, but also because the established diagnostic methods are not always successful in isolating the organism (4). The laboratory confirmation of human brucellosis is based on the microbiological, serological or molecular methods, each having its own advantages and disadvantages. Many serological tests such as ELISA, Standard agglutination test (Wright) and Coombs Wright test are used for the diagnosis of human brucellosis, among which serological tests have a key role in the rapid and proper diagnosis. In addition to the fact that serological tests are easy and safe to use, these tests are quite inexpensive (5, 6). Standard agglutination test, the most common and important serological test, is used for diagnostic purposes in Iran. However, disadvantages of this test include the possibility of false negative results due to the presence of blocking antibodies in addition to the low specificity of the test in endemic area due to the presence of high antibody prevalence in the healthy population. Therefore, in endemic areas, the diagnosis of brucellosis should be confirmed by bacterial isolation which is mostly done by blood culture. This bacterium is very slow growing, requiring seven days or more to grow. The sensitivity of blood culture is usually low (50 - 90% in various studies) depending on the disease stage, *Brucella* species, type of the medium and technique used. Moreover, the risk of transmission of the infection, during blood culture, threatens the laboratory personnel (7, 8).

In the recent years, automated blood culture systems have been developed that reduce the bacterial growth time to a week or less. In case of no access to automated systems for the isolation of *Brucella*, specific media, subculture and prolonged incubation is required. Improved new technologies are not usually available in the rural areas and developing countries (3, 9). Recently, Fatolahzadeh and colleagues have introduced a new medium for the isolation of Brucella, named as TUMS medium. Since in endemic areas, the definitive diagnosis of brucellosis requires isolation of bacteria and TUMS medium is very efficient for the isolation of Brucella from patients, so the use of this medium is very helpful (10).

The development of new diagnostic techniques that facilitate rapid detection and identification of brucellae and minimize the risk of laboratory infection is of great practical importance and it seems that Brucellacapt kit is one of the best options among the new diagnostic methods (11).

Several studies claim that the technology used in the Brucellacapt kit is unique and very useful in detecting both the acute and chronic diseases which is applicable in brucellosis-endemic areas. Other advantages of Brucellacapt test are immediate performance, simple use and handling, simple package kit including all the reagents, no need of washing and easy reading in 24 hours (12, 13). The aim of this study was to evaluate Brucellacapt technique as a diagnostic test for human brucellosis in comparison with (IgG+ IgM) ELISA test.

## MATERIALS AND METHODS

Individuals included in this study were divided into three groups. The first group included 11 patients who had a positive blood culture. The second group included 46 individuals, who had clinical symptoms and signs of brucellosis, but blood cultures were not performed for them and the third group included 32 healthy persons used as control. A total of 89 serum samples were obtained from these three groups and the Standard agglutination test (SAT), Brucellacapt, and ELISA for IgM and IgG antibodies were performed on each serum sample. All the samples from a given patients were processed simultaneously. The Brucellacapt test (Vircell Company, Spain) is based on an immunocapture agglutination method and, in a single step, detects agglutinating antibodies as well as non-agglutinating IgG and IgA antibodies. The test was performed according to the manufacturer's instructions: Briefly, 50 µl of each serum diluents was added to a microplate with U-shaped wells, pre-coated with antihuman immunoglobulin, and then 5 µl of serum samples were added to each well and subsequently 50µl of an antigen suspension (colored *B. melitensis* bacteria killed by formaldehyde treatment) was added to all the wells. The plate was sealed with adhesive tape to avoid evaporation of the liquid in microplate wells and incubated for 18–24 h at 37°C in a dark humid chamber before visualizing the microplate. Positive reactions show agglutination over the bottom of the well. Negative reactions were confirmed by a pellet in the center bottom of the well (13).

SAT test materials were supplied by Pasture Institute (Tehran, Iran), while IgG, IgM ELISA kits were manufactured by Vircell Company, Spain. Briefly, the procedures of these tests are as follows:

The SAT test was performed in tube by a double-dilution method from an initial 1/20 dilution. SAT reactions were read after 24 h incubation at 37°C. The highest serum dilution, showing more than 50% agglutination, was considered the agglutination titer (14).

The IgM and IgG sandwich ELISA tests were performed using the commercial kit. Separate microplates for IgM and IgG were used. The principle of IgM and IgG ELISA was that the plate wells were coated with LPS antigen of *Brucella abortus* to bind corresponding antibodies of the patient sera samples. In the first step, the serum antibodies were bound to the existing antigens in the well. After washing the wells to remove all the unbound antibodies, the anti human IgM or IgG secondary antibody conjugated to the enzyme peroxidase was added. The intensity of the created color, that was in accordance with the amount of serum IgG or IgM, was read in the wavelength of 450 nanometer. An intensity value less than 9 were considered as negative and a value more than 11 as positive (15).

## RESULTS

The efficacy of the two serological tests (ELISA and Brucellacapt), on detecting anti *Brucella* antibodies, was evaluated on a total of 89 serum samples.

In the first group, including 11 patients with a positive blood culture, Brucellacapt, IgM and IgG ELISA tests were positive in 11, 8 and 9 patients, respectively. The IgM and IgG ELISAs failed to detect specific antibodies in 3 and 2 confirmed patients, respectively. On the other hand, Brucellacapt test was positive for all the 11 confirmed patients and its efficacy was equal to the (IgG + IgM) ELISA test. The serum samples from healthy individuals were uniformly detected as negative by the IgM and IgG ELISA tests, while one serum sample, from these healthy individuals, was detected as positive by the Brucellacapt test (Table 1). All samples from the control group were negative by the IgM and IgG ELISA tests (Table 1).

**Table 1:** Results from the sera of brucellosis patients and controls in serological tests.

Group	No. of Persons	Brucellacapt		ELISA/IgM		ELISA/IgG		ELISA (IgG + IgM)	
		POS (%)	NEG (%)	POS (%)	NEG (%)	POS (%)	NEG (%)	POS (%)	NEG (%)
**Positive blood culture**	11	11 (100)	0(0)	8 (72.7)	3(27.3)	9 (81.8)	2(18.2)	11 (100)	0(0)
**Suspected patients**	46	37(80.4)	9(19.6)	15(32.6)	1(67.4)	34(73.9)	2(26.1)	37(80.4)	9(19.6)
**Healthy persons**	32	1(3.2)	31 (96.8)	0(0)	32(100)	0(0)	32(100)	0(0)	32(100)

The sensitivity and specificity of Brucellacapt, IgM ELISA, IgG ELISA and (IgG + IgM) ELISA are summarized in Table 2. To determine the specificity of serological tests, sera from 32 healthy donors were included in this study. No significant difference was established between the specificity of Brucellacapt, (IgG + IgM) ELISAand IgG or IgM ELISA tests. Only one healthy donor had a positive Brucellacapt test (specificity, 96.8%). All the controls were determined as negative by the SAT and ELISA tests (specificity, 100%).

**Table 2:** Sensitivity and Specificity of the Brucellacap and ELISA tests in the sera of 11 confirmed and 46 suspected patients of brucellosis.

	Sensitivity	Specificity
Brucellacapt	80.4	96.8
IgM ELISA	40.6	100
IgG ELISA	75.4	100
(IgM + IgG) ELISA	80.4	100

**Fig. 1:**
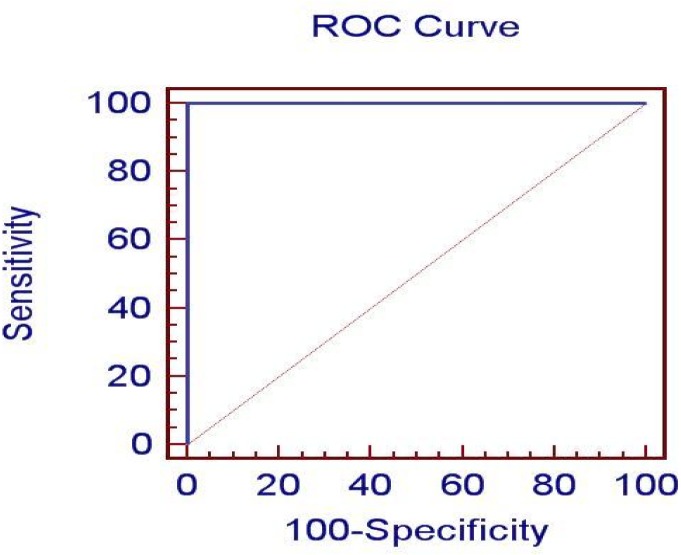
Receiver Operating Characteristic (ROC) curve distinguishing between patients confirmed with brucellosis and those without the disease (control group) diagnosed by IgG ELISA assay.

The area under the ROC curve, for the discrimination of the positive and negative brucellosis groups, was significantly (P < 0.0001) different from 0.5 (Fig. 1). Sensitivity and specificity were calculated for different levels of IgG ELISA. Compared to other cut-off points of IgG ELISA, the cut-off point of 10.78 IU/ml produced the highest sensitivity and specificity and it was, consequently, considered the best cut-off point of IgG ELISA for the diagnosis of human brucellosis. At this point, the sensitivity and specificity were 100 and 100%, respectively (Table 3).

**Table 3:** The power of different IgG ELISA titers in the diagnosis of human brucellosis.

ELISA titers (10.78)	Sensitivity	Specificity
10.78	100	100
12.41	88.89	100
14.19	77.79	100
15.35	66.67	100
16.74	55.56	100

## DISCUSSION

Serological tests have many advantages including simplicity of performing, low cost and no risk of infection for laboratory personnel. However, the fundamental weak spot of serological tests is their low specificity, especially in endemic areas.

In Iran, as an endemic area of brucellosis, the diagnostic values of different serological methods for the diagnosis of human brucellosis have been compared in a few studies. In a study conducted by Vakili *et al*., which included 457 individuals with clinical presentation of brucellosis and a control group with 50 healthy individuals, sensitivity and specificity were found as 93.7% and 70.6% for IgG ELISA and 12.5% and 100% for IgM ELISA, respectively (16).

In another study, Mohraz *et al*., reported that the sensitivity and specificity of IgG ELISA were 93% and 100%, respectively (17). The sensitivity of IgG ELISA in this study was less than that reported in the studies of Mohraz *et al*., (93%) and Vakili *et al*. (93.7%). However, the sensitivity of IgM ELISA in the current study was higher than that in Vakili et al., (12.5%) study. The specificity of IgG ELISA of Mohraz *et al*., study (100%) and IgM ELISA of Vakili *et al*., study (100%) support the result of this study.

According to the literature review, this is the first study in Iran to assess the diagnostic value of Brucellacapt test for the diagnosis of human brucellosis and, therefore, the comparison between the Brucellacapt results with those in other Iranian studies is not possible. Orduna *et al*., compared the sensitivity and specificity of Brucellacapt and coombs tests on the serum samples of 82 definite patients, whose disease was confirmed by blood culture, 157 suspected patients of brucellosis and 412 individuals from people living in rural areas with endemic of brucellosis as control. Their study showed that the Brucellacapt and coombs tests had similar sensitivity and specificity for diagnosis of human brucellosis. They announced that Brucellacapt test is more sensitive and usually showed higher titers than coombs test, although when titers less than 1/320 were used as diagnostic threshold the test specificity slightly decreased (15).

In a study conducted by Ozdemir *et al*., 200 serum samples of suspected patients of brucellosis were assessed and it was found that the sensitivity of Brucellacapt test was similar to (IgG + IgM) ELISA in the means of brucellosis diagnosis. The results obtained by Ozdemir *et al*., are quite consistent with the results of the current study (18).

The difference between the results obtained by ELISA and Brucellacapt tests in various studies can be explained in several aspects. The selection of a gold standard is the most important and difficult step in the studies including diagnostic tests. Some investigators believe that finding a true gold standard is not achievable and for this reason, finding a diagnostic test that is able to completely differentiate between patients and healthy individuals is impossible. However, the establishment of an operational standard for measuring the accuracy of diagnostic tests in all studies is very important and has very influential effects on the results of studies (19).

In Various studies that have been conducted to determine the efficacy of diagnostic tests, different gold standards have been used. Criteria for the selection of patients in the present study were based on a positive blood culture or a positive Wright test ≥1/160, while in the study of Vakili *et al*., patients were selected on a clinical-symptom basis.

It is quite obvious that different criteria for the selection of patients affect the study results. Various antigens used in kits can be another reason for the different results of different studies. In this study, we used ELISA and Brucellacapt kits, manufactured by Vircell Company. Ozdemir et al. used the same kits whilst the ELISA kit used by Vakili *et al*., was manufactured by the IBL Company.

In conclusion, the main drawback of serological tests, in endemic areas, is the low specificity level. Both the ELISA and Brucellacapt tests have demonstrated a high specificity in the current study and this is an advantage for these tests. Based on the results of this study, it can clearly be found that the Brucellacapt and the (IgG + IgM) ELISA tests are valuable tools for diagnosis of brucellosis in Iran as an endemic area of brucellosis.

### Funding

This study was funded by the Vice Chancellor for research at Ardabil University of Medical Sciences.
